# The Influence of Fatigue on Throwing and YBT-UQ Performance in Male Adolescent Handball Players

**DOI:** 10.3389/fspor.2020.00081

**Published:** 2020-07-03

**Authors:** Julian Bauer, Marco Hagen, Nelson Weisz, Thomas Muehlbauer

**Affiliations:** Division of Movement and Training Sciences/Biomechanics of Sport, University of Duisburg-Essen, Essen, Germany

**Keywords:** exhaustion, upper quarter mobility/stability, throwing velocity, young athletes, functional testing

## Abstract

**Purpose:** The goal of the present study was to assess the effects of fatigue on throwing and Upper Quarter Y Balance Test (YBT-UQ) performance in male adolescent handball players. We hypothesized that throwing and YBT-UQ performance will be decreased in response to an upper-body fatigue-protocol.

**Method:** All male participants (*N* = 24, age: 14.8 ± 0.7 yrs) were handball players of a regional youth selection team. A radar gun was used for the assessment of throwing velocity. The YBT-UQ was executed assessing medial, inferolateral and superolateral reach directions normalized to the upper limb length together with a composite score. Immediately following a fatigue protocol of different sets of push-ups until failure (i.e., not being able to perform 60% of the initial maximal amount of push-ups), throwing and YBT-UQ performance were assessed again.

**Results:** Fatigue resulted in a significant decrease in throwing velocity (−3%, *p* = 0.022, *d* = 0.32). Concerning YBT-UQ, the fatigue protocol produced significant decreases for the superolateral reach direction (throwing arm reach: −5%, *p* = 0.017, *d* = 0.39; non-throwing arm reach: −10%, *p* < 0.001, *d* = 0.87) and the composite score (throwing arm reach: −2%, *p* = 0.026, *d* = 0.31; non-throwing arm reach: −4%, *p* = 0.001, *d* = 0.52) but not for the medial and the inferolateral reach directions.

**Conclusions:** Fatigue was found to be an impairing factor for throwing performance and shoulder mobility and stability. Therefore, a lower level of fatigue and/or a higher tolerance of fatigue is desirable. Strength-endurance and mobility exercises especially for the shoulder girdle may be a valuable addition for the training routine of youth handball players.

## Introduction

Fatigue is a physiological and psychological phenomenon experienced to different extents in any kind of physical activity (Ament and Verkerke, 2009). Based on the definition of Ament and Verkerke (2009), fatigue is defined as a state during which the physiological output can only be maintained with higher effort, whereas exhaustion forces the body to refrain from further physiological activities with the same intensities. Neurological, biochemical, biomechanical, and psychological interconnected pathways of fatigue have been proposed (Halperin et al., 2015). Fatigue was demonstrated to be a diminishing factor in maximum force generating capacity, movement coordination, motor control precision, muscle reaction times, and proprioception in sporting activities (Hakkinen and Komi, 1986; Skinner et al., 1986; Parnianpour et al., 1988; Sparto et al., 1997; Madigan and Pidcoe, 2003). Whereas local fatigue mainly concerns the primary movers, whole body fatigue is characterized by a multi-system involvement (Wassinger et al., 2014) that may also affect upper and lower extremities not involved as primary movers (Cetin et al., 2008), with a decline in force production at or distal to the neuromuscular junction (Gandevia, 2001; Taylor and Gandevia, 2008).

As sport performances are often executed under fatigued conditions, different functional tests have been proposed. As one of the assessment tools, the Upper Quarter Y Balance Test (YBT-UQ) is a test requiring both shoulder and core stability while performing arm movements in three different directions in a closed kinetic chain position (Gorman et al., 2012). Previous studies on the influence of fatigue on YBT-UQ performance have investigated healthy young adults, only. For example, Salo and Chaconas (2017) examined fatigue to be a diminishing factor in YBT-UQ scores stating a reduction of 2.04 to 12.16 cm in different directions after a shoulder press, prone push-up, and pull up exercises to failure protocol by adults regularly performing weight training. Also handball-related abilities are reported to be significantly decreased by fatigue. It is reported that maximal and rapid force development are negatively affected following simulated match-play actions, leading to impaired match performance (Thorlund et al., 2008). As a result of fatigue, decreased handball-specific performance with less high-intensity actions has been reported in the second half of elite handball games (Michalsik et al., 2015). For throwing actions this may be due to diminished somatosensory information in the shoulder following local fatigue of the upper extremities (Niederseer et al., 2014). Additionally, throwing kinematics and kinetics may be negatively influenced by fatigue. Throwing velocity may decrease as fatigue increases (Nuno et al., 2016). As throwing is reported to be influenced by muscle strength (Andrade et al., 2016), it can be expected that a decreased muscular output following fatigue will lead to a diminished throwing velocity. However, transferring these results obtained from young adults to adolescents appears to be questionable, given that anthropometric and physiological differences due to growth and maturation exist (Malina et al., 2004). As a consequence, performance decrements achieved following fatigue may differ between age groups.

The primary purpose of the present study was to analyse whether there are pre- to post-fatigue differences in throwing velocity. The secondary purpose was to analyse whether there are decreases in upper quarter mobility and stability performance as assessed through the YBT-UQ. With reference to the relevant literature (Thorlund et al., 2008; Salo and Chaconas, 2017; Michalsik, 2018), we expected decreases in throwing and YBT-UQ performance in youth handball players following an upper-body fatigue-protocol.

## Method

### Participants

All participants played in a regional youth selection team of the Handball Association Niederrhein who had a comparable training regimen in terms of quantity (3–4 times per week) and similar playing level (Table 1). In terms of the league level, all players perform at the highest regional level, as in this age category no national league exist. The players and their parents were informed about the study procedure, testing protocol, and possible risks. Written consent of all players and informed consent of the parents or legal guardians was obtained before the testing. Most players already executed the throwing and YBT-UQ before and were familiar with the testing procedure. The human ethics committee of the Faculty of Educational Sciences, University of Duisburg-Essen, Germany approved the study (TM_23.03.2020) and it was executed in line with the Declaration of Helsinki.

**Table 1:** Characteristics of the study participants.

**Characteristic**	**Male handball players (*N* = 24)**
Age [years]	14.8 ± 0.7 (14.0–16.0)
Body height [cm]	180.4 ± 6.4 (162.5–191.5)
Body mass [kg]	70.3 ± 7.9 (58.4–88.5)
Body mass index [kg/m^2^]	21.6 ± 2.2 (19.1–26.2)
Non-throwing arm length [cm]	91.6 ± 4.1 (81.5–99.0)
Throwing arm length [cm]	92.3 ± 3.9 (82.5–97.0)
Training experience [years]	7.0 ± 2.6 (3.0–11.0)
Throwing arm [L/R]	2/22

*Values are mean values ± standard deviations and range in parentheses. L, left; R, right*.

### Testing Procedures

#### Measurement

**Figure 1:**
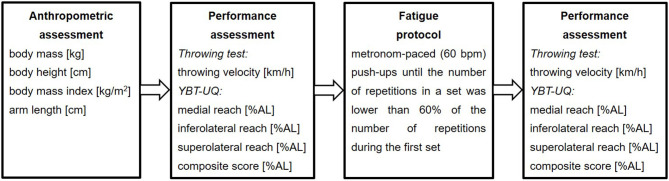
Schematic description of the study design. AL, arm length; YBT-UQ, Upper Quarter Y Balance Test.

The testing took place at a regular training session in the evening. Testing personnel was made up of experienced raters who were familiar with every testing station. The players were randomly and equally assigned to the three groups for the three testing stations (anthropometric assessment; YBT-UQ; throwing velocity) (Figure 1). The three testing stations were performed consecutively by every group in a different order. Following the first three testing stations, the three groups were divided into two groups. These two groups subsequently performed the fatigue protocol and the tests on both sides and both goals of the training venue. A standardized verbal instruction was given prior to the tests with a warm-up of 5 min of submaximal running followed by a mobility routine of different functional exercises. Prior to the throwing test, different standardized passing techniques (with handballs of the according throwing size 2) were executed.

#### Fatigue Protocol of the Upper Body

Based on a metronome paced with 60 bpm, a push-up to failure protocol (Salo and Chaconas, 2017) was executed by every individual player as the fatigue stimulus. The lifting phase was 1 s, with the lowering phase also being 1 s, making the whole cycle of eccentric and concentric phase being 2 s. Based on this maximal number of repetitions (100%), a 60% value was calculated as proposed by Lesinski et al. (2016). After a rest of 1 min, the players had to execute the push-up exercises again with the same pace (60 bpm) until exhaustion. If at least 60% of the maximum repetitions were executed, the subject again had a 1 min break before executing the push-ups in the same way for another time. This protocol was executed until the subject was not able to perform at least 60% of the maximum number of repetitions. The push-up protocol was executed immediately following the warm-up routine. After the break-up (i.e., not being able to perform at least 60% of the initial numbers of push-ups), the subjects went to the throwing station and threw onto the target net, leading to ~10 s between the break-up of the push-ups and the throwing.

#### Assessment of Anthropometric Characteristics

Upper limb length was measured from the seventh cervical spinous process to the distal tip of the middle finger with the shoulder being in a 90° extension (Teyhen et al., 2014). Body mass was assessed with a Seca clara 803 digital scale. Standing body height was assessed without shoes with a Seca linear measure scale. All subjects were asked to look forward and standing straight leaning against the scale. Body height was assessed in centimeters from the ground to the top of the subjects' head. Playing and training experience was assessed in years and the players were asked which position they play and what their throwing arm is.

#### Assessment of Throwing Velocity

The throwing was executed pre- and directly post-fatigue. A target net (SG 500L; size: 3 m × 2 m) was attached to both handball goals. Throwing velocity was assessed using a “Stalker Pro” radar gun (Applied Concepts Inc., TX, USA). The radar gun is able to measure velocities from 0 to 480 km/h with an accuracy of 0.16 km/h in a 0.01 s time interval. The working frequency of the “Stalker Pro” is 35.1 GHz with a low disturbance threshold. The radar gun was positioned behind the goal net in the height of 1.20 m facing through the hole of the goal net to secure the Doppler effect. One tester was constantly positioned behind the camera and registered the values while a second one noted down the values into the score sheet of the individual athlete. During throwing the contralateral leg was positioned at the 7-m line while the throwing arm held the ball. The contralateral foot was allowed to touch the bench that was positioned in front of the 7 m line. The participants were not allowed to fall over the bench after the throw. All athletes used a standard ball size 2 and glue or resin was allowed to simulate a game- or training-like situation. Each player had three consecutive trials immediately following each other and only the best trial (fastest velocity) was counted. As throwing onto the goal is usually only executed with the throwing arm, only this arm was tested.

#### Assessment of Upper Quarter Y Balance Test Performance

The YBT-UQ was executed with a Y Balance Test Kit (Move2Perform, Evansville, USA) with two adapted YBT-UQ test protocols (pre- and post-fatigue condition) together with columns to assess the maximal number of push-ups executed, the 60% value of the maximal push-ups and number of sets performed until failure. A standardized verbal instruction was given to all participants prior to the tests and one of the experimenters demonstrated the test procedure before the start. The participants placed their dominant hand at the center of the junction and reached out to the furthest point of each direction with the free hand in medial, superolateral, and inferolateral direction without a break and while maintaining the stabilization with the arm tested. Trials were stopped if the participants did not maintain three-point contact or touched the floor with the mobile arm or hand (Gorman et al., 2012). Thirty seconds breaks were granted after each trial prior to the next trial. After finishing all three reach directions of the first arm, the participants executed the same protocol for the other arm. The best score (i.e., maximal reach distance) was noted down for every direction and limb tested with reaches normalized for upper limb length and an additional normalized composite score (CS) was calculated as the mean of the averaged maximal distances in all reach directions.

### Statistical Analyses

Data are presented as group mean values ± standard deviations. After data were tested for normal distribution (i.e., Shapiro-Wilk test with *p* > 0.05), a paired *t*-test was applied to analyze fatigue-related performance changes. In addition, effect size (Cohen's *d*) was determined and classified as small (0 ≤ *d* ≤ 0.49), medium (0.50 ≤ *d* ≤ 0.79), and large (*d* ≥ 0.80). All analyses were performed using Statistical Package for Social Sciences (SPSS) version 24.0 (SPSS Inc., Chicago, Illinois, USA). The significance level was set at *p* < 0.05.

## Results

### Fatigue Protocol

**Figure 2:**
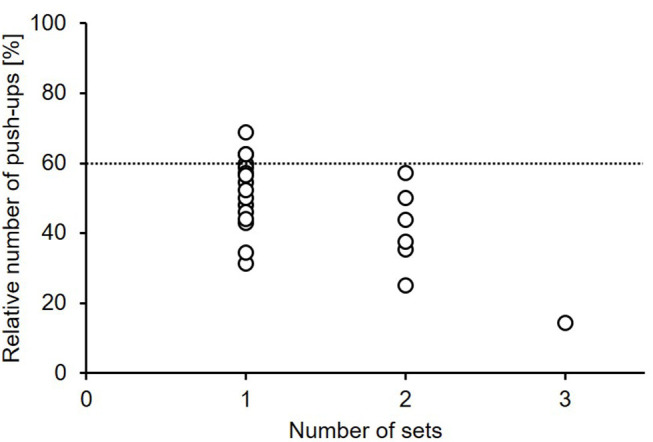
Relative number of push-ups until fatigue-related task failure. Note: The metronome-paced (60 bpm) push-ups were performed until the number of repetitions in a set was lower than 60% (indicated by the dotted line) of the number of repetitions during the first set (i.e., 100%).

After determining the maximal number of push-ups (equals 100%), all players performed a first set of push-ups until task failure. Six out of 24 players achieved the minimum of 60% or higher from the maximum and performed a second set of push-ups until failure (Figure 2). Only one player reached the minimum of 60% again and conducted a third set.

### Effect of Fatigue on Throwing and Upper Quarter Y Balance Test Performance

In terms of throwing performance, fatigue resulted in a significant decrease in throwing velocity (−3%, *p* = 0.022, *d* = 0.32) (Table 2). Concerning YBT-UQ, the fatigue protocol produced significant decreases for the superolateral reach direction (throwing arm reach: −5%, *p* = 0.017, *d* = 0.39; non-throwing arm reach: −10%, *p* < 0.001, *d* = 0.87) and the CS (throwing arm reach: −2%, *p* = 0.026, *d* = 0.31; non-throwing arm reach: −4%, *p* = 0.001, *d* = 0.52) but not for the medial (throwing arm reach: −2%, *p* = 0.148, *d* = 0.23; non-throwing arm reach: −1%, *p* = 0.298, *d* = 0.16) and the inferolateral reach directions (throwing arm reach: −1%, *p* = 0.637, *d* = 0.08; non-throwing arm reach: ±0%, *p* = 0.757, *d* = 0.04) (Table 2).

**Table 2:** Variables of throwing and Upper Quarter Y Balance Test performance separated by fatigue.

**Measure**	**Non-fatigued**	**Fatigued**	**Δ, %**	***p*-/*d*-value**
Throwing velocity [km/h]	84.5 ± 7.2	82.3 ± 6.6	−3	0.022/0.32
**Throwing arm reach**				
MD [% AL]	109.5 ± 8.9	107.6 ± 8.0	−2	0.148/0.23
IL [% AL]	109.2 ± 11.6	108.2 ± 12.28	−1	0.637/0.08
SL [% AL]	84.8 ± 11.8	80.4 ± 10.8	−5	0.017/0.39
CS [% AL]	101.2 ± 8.3	98.7 ± 7.5	−2	0.026/0.31
**Non-throwing arm reach**				
MD [% AL]	111.6 ± 9.5	110.2 ± 8.5	−1	0.298/0.16
IL [% AL]	110.5 ± 10.6	110.1 ± 9.3	0	0.757/0.04
SL [% AL]	89.1 ± 10.2	79.9 ± 11.2	−10	<0.001/0.87
CS [% AL]	103.7 ± 7.2	100.0 ± 7.3	−4	0.001/0.52

*Values are means ± standard deviations. AL, arm length; CS, composite score; IL, inferolateral; MD, medial; SL, superolateral; d, effect size*.

## Discussion

To the authors' knowledge, this is the first study to examine the effect of fatigue on throwing and YBT-UQ performance in healthy young male handball players. The main result of this study was that fatigue produced significantly lower throwing velocity and YBT-UQ reach distances.

### Effect of Fatigue on Throwing Velocity

In accordance with our hypothesis of fatigue-related decrements in throwing performance, we found that throwing velocity was significantly lower after the upper-body fatigue protocol. Based on the results, the present level of fatigue impaired the testing scores. The present results are in line with findings of previous studies. For example, Niederseer et al. (2014) reported a decrease in the joint position sense of handball players following a simulated handball match while Bedo et al. (2020) stated reductions in maximum propulsion force and center of pressure. On a biomechanical level, higher stabilizing function of the glenohumeral joint in the fatigued state (Teyhen et al., 2008) which may lead to lower muscle output and decrease in force production might be a possible explanation. As the present protocol had a local fatigue effect, the decline in force production at or distal to the neuromuscular junction (Wassinger et al., 2014) may be the responsible neuromuscular process for the decreased muscular output leading to lower throwing velocity. Muscle fatigue may desensitize the muscle spindle threshold through metabolic changes (e.g., lactic acid) (Pedersen et al., 1999) on the biochemical level. Metabolic by-products from the fatigued muscles may have been distributed to remote muscles and may have hindered contractile ability (Halperin et al., 2015).

### Effect of Fatigue on Upper Quarter Y Balance Test Performance

Partly in line with our assumption of fatigue-related impairments in upper quarter mobility/stability, the YBT-UQ values for the throwing and non-throwing arm were significantly decreased after the fatigue protocol for the superolateral reach direction and for the CS. This finding goes in line with Salo and Chaconas (2017) who reported significantly lower YBT-UQ scores following shoulder press, machine row, prone push-up, and pull-up exercises. However, contrary to the protocol of Salo and Chaconas (2017), in the present study, push-ups until fatigue alone already induced a sufficient stimulus to decrease YBT-UQ values. No short-term inter-exercise recovery might have taken place for the muscles mainly involved in the throwing action. The peripheral reduction in neural drive that subsequently also affects the neural drive of the rested or stabilizing muscles (Halperin et al., 2015) may have led to the decreased performance of the throwing and non-throwing side. Peripheral neural fatigue may have additionally activated type 3 and type 4 muscle afferents that led to the inhibition of the central neural drive of spinal and cortical motoneurons (Behm, 2004). The significantly decreased CS of both arms seems to be a consequence of the strong impact and effect size of the superolateral reach direction results, which in combination with the medial and inferolateral direction compromises the CS.

The YBT-UQ test was executed following the fatigue protocol and the throwing task. The performance for the superolateral but not for the medial and the inferolateral reach direction was significantly impaired following the fatigue protocol. A possible reason might be that the superolateral reach direction was the last one being tested in the sequence of the three reach directions leading to the longest hold-up time in the weight bearing one-arm push-up position which could have negatively affected the recovery process. Furthermore, the superolateral reach direction most closely resembles the overhead throwing technique (Wilson et al., 2013). Thus, the throwing task could had an additional detrimental effect on the subsequently performed YBT-UQ, particularly with regard to the superolateral reach direction.

### Limitations

There are a few limitations with this study that need to be addressed. First, we investigated male adolescent handball players, which limits the transferability of our findings to younger (children) and older (adults) players as well as to female players of different ages. Thus, future studies are advised to include individuals from other age groups and of both sexes. Second, our finding of fatigue-related impairments on throwing and YBT-UQ performance is limited to the push-up protocol that was used to induce upper-body fatigue. Future studies should investigate whether this can be transferred to a more real-sports scenario (e.g., throwing protocol). Third, we did not apply electrophysiological measurements and therefore can only speculate on the underlying mechanisms of the observed performance decrements. Especially, with respect to the fatigue-effects on the superolateral reach direction, the application of surface EMG might have provided deeper insights and should be additionally used in future studies.

## Conclusions

The present study investigated the effects of fatigue on throwing and YBT-UQ performance in young, male handball players. We found that fatigue of the shoulder girdle induced by repetitive push-ups until failure lead to deteriorations of throwing velocity and YBT-UQ reach distances (i.e., superolateral reach direction; CS). Because handball is characterized as a game with a high number of low and high intensity intervals, a lower level of fatigue and reliance on glycolytic pathways or a higher tolerance of fatigue is desirable (Michalsik, 2018). Accompanying training diagnostics in the preparatory period, as well as competitive period focusing on stability and mobility especially of the upper-limbs and closed kinetic chain should also be part of functional assessments.

Previous studies have shown that healthy young adults showed worse results in throwing performance and YBT-UQ in fatigued conditions. The present study indicates that this detrimental effect is also present in well-trained adolescent handball players. Therefore, coaches should develop training programs with the goal of either delaying the demonstrated fatiguing effects or establishing a higher fatiguing tolerance. Further, our finding of fatigue-related decrements in throwing shoulder stability and mobility performance indicates that practitioners should develop sophisticated substitution strategies to avoid this performance-diminishing level of local fatigue during game situations.

## Data Availability Statement

The raw data supporting the conclusions of this article will be made available by the authors, without undue reservation.

## Ethics Statement

The studies involving human participants were reviewed and approved by Ethikkommission des Instituts für Psychologie der Fakultät für Bildungswissenschaften der Universität Duisburg-Essen. Written informed consent to participate in this study was provided by the participants' legal guardian/next of kin.

## Author Contributions

TM and JB designed the research question and analyzed the data. JB planned and supervised the testings and wrote the main part of the manuscript. JB, MH, and NW conducted the testings and data collection. TM and MH reviewed the manuscript. All authors approved the final manuscript. All authors contributed to the article and approved the submitted version.

## Conflict of Interest

The authors declare that the research was conducted in the absence of any commercial or financial relationships that could be construed as a potential conflict of interest.
